# Design and Analysis of a Large Mode Field Area and Low Bending Loss Multi-Cladding Fiber with Comb-Index Core and Gradient-Refractive Index Ring

**DOI:** 10.3390/s23115085

**Published:** 2023-05-26

**Authors:** Yining Zhang, Yudong Lian

**Affiliations:** 1 Center for Advanced Laser Technology, Hebei University of Technology, Tianjin 300401, China; 202301@stu.hebut.edu.cn; 2 Hebei Key Laboratory of Advanced Laser Technology and Equipment, Tianjin 300401, China; 3 Tianjin Key Laboratory of Electronic Materials and Devices, Tianjin 300401, China

**Keywords:** large mode field area, low bending loss, gradient-refractive index ring, comb-index core, multi-cladding

## Abstract

The large mode field area fiber can raise the tolerance of power, and high requirements for the bending characteristics of optical fibers are needed. In this paper, a fiber composed of a comb-index core, gradient-refractive index ring, and multi-cladding is proposed. The performance of the proposed fiber is investigated by using a finite element method at a 1550 nm wavelength. When the bending radius is 20 cm, the mode field area of the fundamental mode can achieve 2010 μm^2^, and the bending loss is reduced to 8.452 × 10^−4^ dB/m. Additionally, when the bending radius is smaller than 30 cm, there are two variations with low BL and leakage; one is a bending radius of 17 cm to 21 cm, and the other is from 24 cm to 28 cm (except for 27 cm). When the bending radius is between 17 cm and 38 cm, the highest bending loss is 1.131 × 10^−1^ dB/m and the lowest mode field area is 1925 μm^2^. It has a very important application prospect in the field of high-power fiber lasers and telecom applications.

## 1. Introduction

With the improvement of fiber laser power [1,2,3], some physical mechanism damage limits the development of high-power fiber lasers, including optical damage, transversal mode instabilities, and so on [4,5,6]. Large mode field area (MFA) fiber is an effective way to solve the above problems, and it can improve the capacity of communication [7]. However, the bending loss (BL) is prone to increase with MFA, so there is a contradiction between the BL and MFA [8]. Additionally, with the rapid development of fiber for home technology, higher and higher requirements are put forward for the bending characteristics of optical fibers. During installation and laying, the distributing network density of optical fibers is extremely high, and the laying lines are very complex. Therefore, the investigation of optical fiber with a low BL and large MFA is of significance.

Compared with photonic crystal fiber (PCF) [9,10,11], segmented cladding fiber (SCF) [12,13,14], and multi-core fiber (MCoF) [15,16,17], multi-cladding fiber (MClF) has more advantages. PCF is easy to realize in single-mode and with a low BL [18]. However, because of its multiple air holes, the production process of PCF is complex and the fiber is easy to collapse. MCoF can be used for space-division multiplexing [19,20,21], and its transmission capacity is several times that of single-core optical fiber [22], but it causes varying degrees of inter-core crosstalk [23]. As for SCF, it has a large MFA and high-quality output beam [14], but it also requires a complex manufacturing process, and the process is difficult to control. More current statuses of similar research is shown in Table 1. In contrast, MClFs has advantages over optical fibers for solving the above problems [24]. They are mostly all-solid and symmetrical structures, which greatly simplifies the manufacturing process. Additionally, comb-index fiber (CIF) is beneficial to large MFAs [25], and the gradient-refractive index ring (GRIR) can contribute to improving the MFA and decreasing the BL [26].

In this paper, we proposed a large-MFA and low-BL MClF with a comb-index core (CIC) and GRIR. It combines the advantages of MClF, CIF, and GRIR, and achieves an MFA of 2010 μm^2^ and a BL of 8.452 × 10^−4^ dB/m at a bending radius of 20 cm. The proposed fiber shows excellent performance and is expected to be used in a high fiber laser, fiber for the home, and so on. Additionally, by consulting relevant references on fiber manufacturing, we believe that the proposed fiber can be manufactured with existing technology [1,30].

## 2. Materials and Methods

This section introduces the designed fiber structure and parameters, and then introduces the methods for analyzing its related characteristics.

### 2.1. Structure

**Figure 1:**
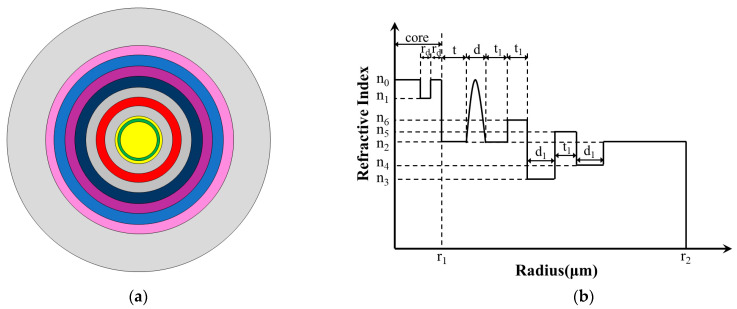
Schematic diagram of proposed fiber; (**a**) 2D cross section; (**b**) RIP.

Figure 1 shows the 2D cross section and refractive index profile (RIP) of the proposed fiber. It indicates that the optical fiber structure is mainly composed of three parts: CIC (yellow area and green area), GRIR (red area), and multi-cladding (blue area, dark blue area, pink area, and purple area).

The fiber proposed has a total of 11 layers, and the refractive index of the yellow, green, grey, red, dark blue, purple, blue, and pink regions is *n*_0_, *n*_1_, *n*_2_, *n_r_*, *n*_6_, *n*_3_, *n*_5_, and *n*_4_, respectively. CIF contributes to achieving a large MFA, so the fiber core is designed as a three-layer comb-index core; the width of the outermost two layers layer is *r_d_* and the radius of the core is *r*_1_. The width of the other layers is *t*, *d*, *t*_1_, *t*_1_, *d*_1_, *t*_1_, *d*_1_, and the radius of the fiber is *r*_2_.

Compared with the step-index resonant rings and trapezoidal-index resonant rings proposed previously, the GRIR can realize more outstanding abilities of low BL and large MFA [31]. Its highest refractive index is *n*_0_, and the RIP can be expressed as follows:

(1) $$n_{r}=n_{0}{\left[1-2∆{\left(\frac{\left|r\right|-d_{0}}{d/2}\right)}^{\alpha }\right]}^{1/2}$$

(2) $$d_{0}=r_{1}+t+\frac{d}{2}$$

(3) $$∆=\frac{n_{0}^{2}-n_{2}^{2}}{2n_{0}^{2}}$$

where *n*_2_ is the lowest refractive index of the GRIR, *r* is the radius of the position, ∆ is the relative refractive index difference, and *α* is the refractive index distribution constant, set as 2 in this paper.

### 2.2. Analysis Methods

The designed fiber in this paper adopts COMSOL Multiphysics simulation software, which is based on the finite element method [32]. The finite element method is dividing a region into several interconnected grids, each of which is represented by a partial differential equation, then obtaining equations and solving them to acquire model parameters. A perfectly matched layer is added outside the cladding, which is used to calculate BL accurately. The perfectly matched layer is an absorbing layer with a certain thickness, and it can completely absorb the incident light [33,34]. The width of the perfectly matched layer in the paper is 8 μm.

The MFA of optical fiber represents the concentrated density of a light wave, and the effective MFA (*A_eff_*) can be expressed as follows [35]:

(4) $$A_{eff}=\frac{{\left(\iint {\left|E\right|}^{2}dxdy\right)}^{2}}{\iint {\left|E\right|}^{4}dxdy}$$

where *E* is the transverse electric field component of fiber, which is related to the optical input wavelength and structural parameters of fiber.

The distortion of the refractive index occurs when the fiber is bent. Hence, the RIP can be expressed with an equivalent formula [36]:

(5) $$n^{*}=n\sqrt{\left(1+\frac{2x}{r_{bend}}\right)}$$

where *n* is the refractive index in the straight state, *x* is the abscissa, *r_bend_* is the bending radius, and n* is the refractive index in the bending state.

The *BL* of the fiber can be written as follows [37]:

(6) $$BL=-\frac{20\pi }{ln10}Im\left(\beta \right)\approx -8.686\frac{2\pi }{\lambda }Im(n_{eff})$$

where *Im(neff)* is the imaginary part of the effective mode-refractive index of the fundamental mode (FM) and *β* is the propagation constant.

## 3. Numerical Simulations

**Figure 2:**
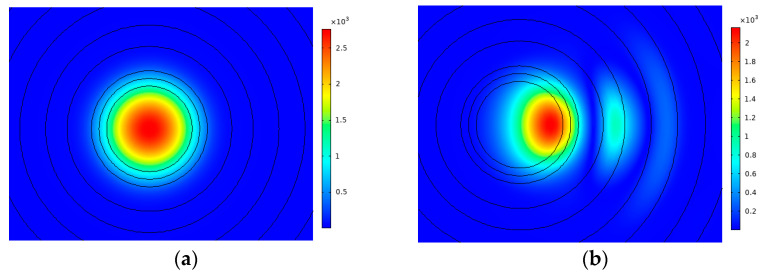
Mode field distribution of FM in (**a**) straight state; (**b**) bending state.

In this section, the performance of the designed fiber is simulated by changing the relevant parameters. The original parameters were set as *n*_0_ = 1.44, *n*_1_ = 1.4398, *n*_2_ = 1.4395, *n*_3_ = 1.4385, *n*_4_ = 1.4386, *n*_5_ = 1.4396, *n*_6_ = 1.4397, *r*_1_ = 38 μm, *r*_2_ = 210 μm, *r_bend_* = 20 cm (*r_bend_* is the bending radius), *r_d_* = 5 μm, *t*_1_ = 17 μm, *d*_1_ = 18 μm, *t* = 16 μm, and *d* = 14 μm, respectively. Unless otherwise stated, the MFA and BL mentioned in the paper are all of the FMs, and the parameters are unchanged. The mode field distribution of the FM in straight and bending states is shown in Figure 2a,b, respectively. Additionally, we define a ratio of BL to MFA to find the best performance.

### 3.1. Numerical Simulations of t_1_ and d_1_

**Figure 3:**
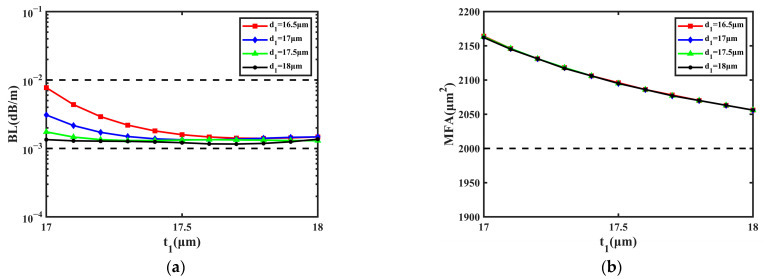
The influence on BL and MFA with the change in *t*_1_ and *d*_1_. (**a**) BL of FM. (**b**) MFA of FM.

First, the effect of the multi-cladding was studied by varying the values of *t*_1_ and *d*_1_; the range of *t*_1_ was 17~18 μm and that of *d*_1_ was 16.5~18 μm. The change in *t*_1_ and *d*_1_ has an excessive effect on the BL, which can lead to intense and irregular fluctuations in the BL, so the small variation range was selected to make the BL remain steady. Figure 3 shows the influence of the cladding’s size on the BL and MFA when *d*_1_ is 16.5 μm, 17 μm, 17.5 μm, and 18 μm, respectively.

From Figure 3a, it can be learned that BL decreases with *t*_1_ and *d*_1_ at first, then becomes flat. Within the variation range of *t*_1_ and *d*_1_, most values of BL are close to 1.000 × 10^−3^ dB/m, and the lowest of them is 1.160 × 10^−3^ dB/m when *d*_1_ is 18 μm and *t*_1_ is 17.7 μm. The BL nearly remains unchanged when *t*_1_ changes from 17.5 μm to 18 μm, so the proposed fiber can obtain a steady performance of the BL. In Figure 3b, it can be considered that MFA has no relationship with *d*_1_ and goes down about 100 μm^2^ with *t*_1_. During the increase in *t*_1_ from 17 μm to 18 μm, the MFA decreases from 2164 μm^2^ to 2056 μm^2^. With the change in *t*_1_, the MFA is still greater than 2000 μm^2^ and can reach the maximum of 2235 μm^2^ when *d*_1_ is 16.5 μm and *t*_1_ is 17 μm. In conclusion, the BL and MFA can remain steady when *t*_1_ changes from 17 μm to 18 μm and *d*_1_ changes from 16.5 μm to 18 μm.

### 3.2. Numerical Simulations of t and d

**Figure 4:**
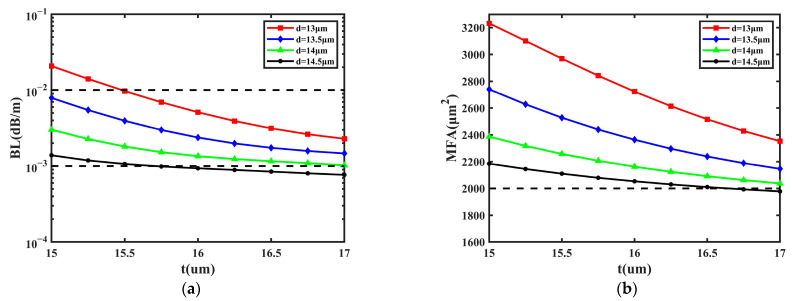
The influence on BL and MFA with the change in *t* and *d*. (**a**) BL of FM; (**b**) MFA of FM.

Second, the impact of the GRIR was also taken into consideration. The simulations were performed by changing the values of *t* and *d*; the range of *t* was 15~17 μm and that of *d* was 13~14.5 μm. The main function of the GRIR is increasing the MFA via coupling [38]. Due to the coupling effect of the ring, the mode field distribution of the FM will gradually transit to the outer layer when the GRIR is added to the fiber, so the MFA can be increased. In Figure 4, the change in the BL and MFA are shown with the values of *d* at 13 μm, 13.5 μm, 14 μm, and 14.5 μm, respectively.

It can be learned from Figure 4a that BL decreases with *t* and *d*, the lowest BL is 7.665 × 10^−4^ dB/m when *d* is 14.5 μm and *t* is 17 μm, and the highest values of BL are less than 1.000 × 10^−2^ dB/m. The BL can be reduced by one order of magnitude with the increase in *d* from 13 μm to 14.5 μm. Additionally, the change in *t* and *d* has a significant effect on the MFA from Figure 4b. The MFA decreases more slowly with *t* and *d*, and the largest MFA achieves 3232 μm^2^ when *d* is 13 μm and *t* is 15 μm; the variety range of MFA is about 1200 μm^2^. Additionally, the ratio of BL to MFA is the lowest when *d* is 14.5 µm and *t* is 16.5 µm, and the BL is 8.452 × 10^−4^ dB/m and the MFA is 2010 µm^2^. As a conclusion, the GRIR can affect the MFA and BL; both the MFA and BL are inversely proportional to *t* and *d*.

### 3.3. Numerical Simulations of r_1_ and r_d_

**Figure 5:**
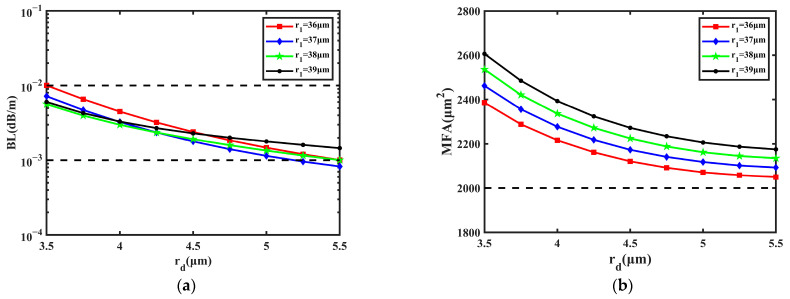
The influence on BL and MFA with the change in *r_d_* and *r*_1_. (**a**) BL of FM; (**b**) MFA of FM.

Next, the impact of the CIC is shown in Figure 4. Theoretically, CIC is beneficial for a large MFA and low BL. In Figure 5, the influence of core size on the BL and MFA is shown when *r*_1_ is 36 μm, 37 μm, 38 μm, and 39 μm, respectively. The simulations were performed by changing the values of *r*_1_ and *r_d_*, and the range of *r_d_* was 3.5~5.5 μm.

From Figure 5a, it can be learned that the BL decreases with *r_d_*; the lowest BL is 8.250 × 10^−4^ dB/m when *r_d_* is 5.5 μm and *r*_1_ is 37 μm. Only when *r_d_* is 3.5 μm and *r*_1_ is 36 μm can the BL be larger than 1.000 × 10^−2^ dB/m. As for *r*_1_, it can be regarded as having no significant effect on the BL. From Figure 5b, the MFA decreases and then becomes steady with *r_d_* and *r*_1_. The largest MFA is 2606 μm^2^ when *r_d_* is 3.5 μm and *r*_1_ is 39 μm, and the MFA is still larger than 2000 μm^2^. It can be concluded that with the increase in *r_d_*, the BL can be reduced by one order of magnitude, and the variation range of the MFA is close to 600 μm^2^.

## 4. Comparison and Analysis

As mentioned in Section 3, the proposed fiber consists of three parts: CIC, GRIR, and multi-cladding. To further confirm the function of each part, a comparison of the three parts was performed, respectively.

### 4.1. Importance of the CIC

**Figure 6:**
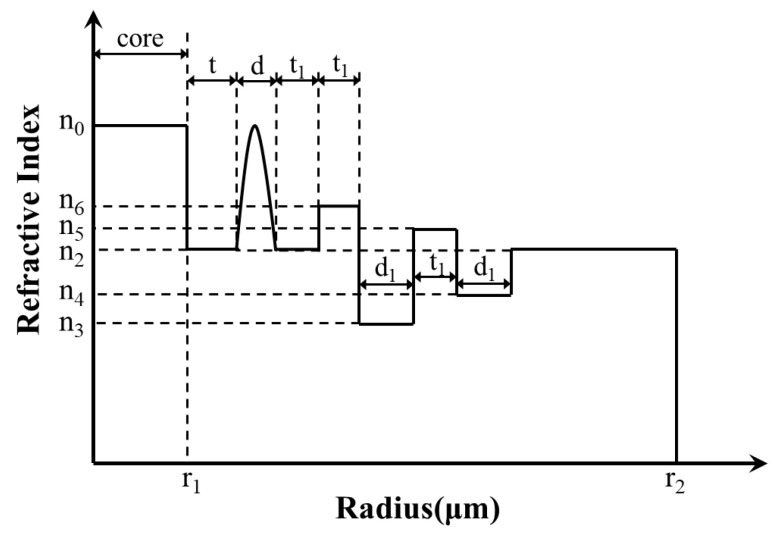
The RIP of the SIC fiber with multi-cladding and a GRIR.

**Figure 7:**
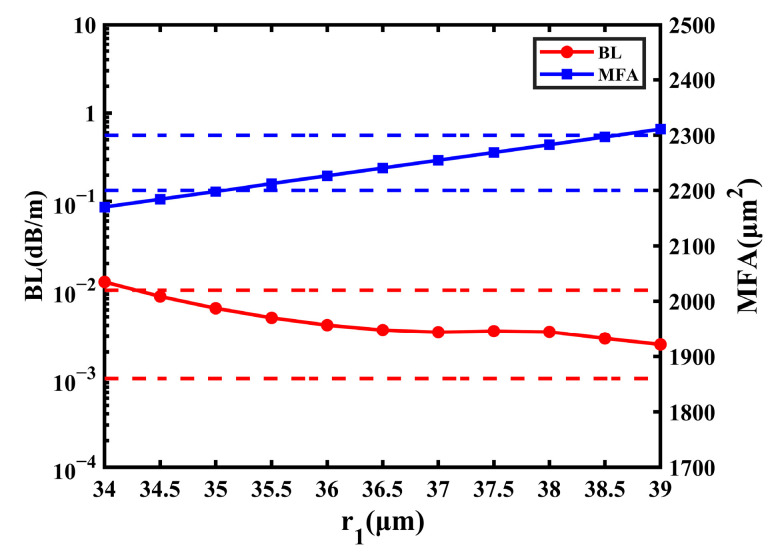
The influence on BL and MFA of the SIC fiber with the change in *r*_1_.

Figure 6 shows the RIP of the fiber with a step-index core (SIC). Compared with the structure in Figure 1, the only difference is that the core changes from comb-index to step-index. Additionally, the fiber with an SIR cannot support the transmission of the FM when the bending radius is 20 cm, so the radius needs to be increased to at least 24 cm to support the FM. The change in the BL and MFA are shown in Figure 7 with a range of *r*_1_ from 34 μm to 39 μm.

From Figure 7, it can be learned that the BL decreases a little and tends to flatten out, and the lowest BL is 2.425 × 10^−3^ dB/m. Compared with the fiber with the CIC, the BL of the SIC structure rises slightly, in general. Additionally, with the increase in *r*_1_, the MFA increases slightly and it changes from 2170 μm^2^ to 2311 μm^2^ in Figure 7. When *r*_1_ is 39 μm, the MFA of the fiber with the CIC can achieve 2606 μm^2^. Compared with this, the largest MFA of the fiber with an SIC decreases by 295 μm^2^. In conclusion, the SIC is beneficial to improve the performance of fiber by reducing the BL and increasing MFA to some extent, and the CIC can allow the smaller bending radius to transmit the FM.

### 4.2. Importance of Multi-Cladding

**Figure 8:**
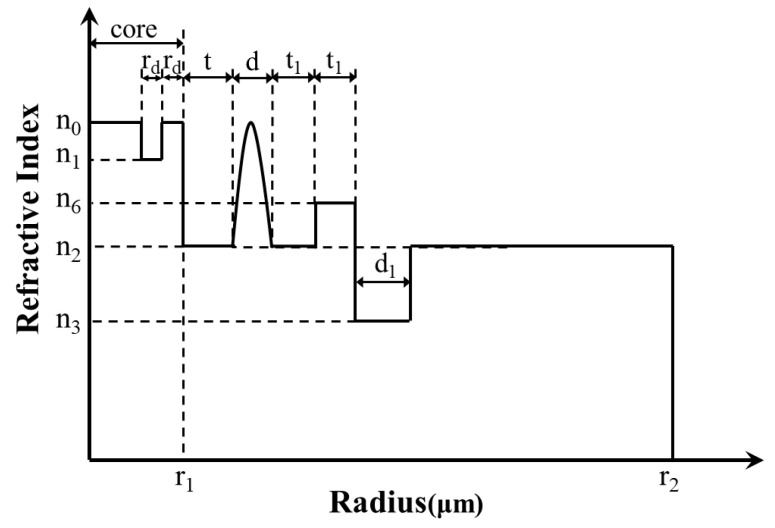
The RIP of the three-cladding fiber with CIC and a GRIR.

**Figure 9:**
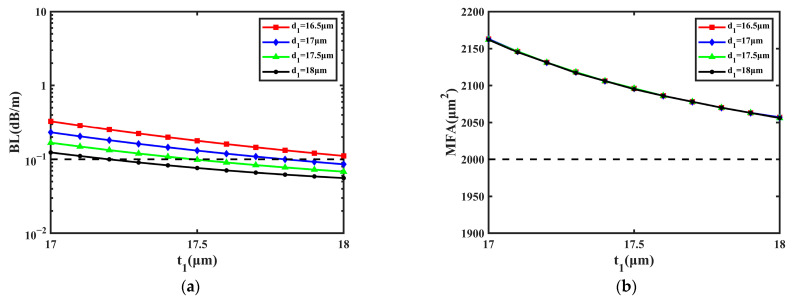
The influence on BL and MFA of the three-cladding fiber with the change in *t*_1_ and *d*_1_. (**a**) BL of FM; (**b**) MFA of FM.

Figure 8 shows the RIP of the CIC fiber with three cladding and a GRIR. Compared with the structure in Figure 1, the only difference is that the cladding changes from five-cladding to three-cladding. The range of *t*_1_ is 17~18 μm and that of *d*_1_ is 16.5~18 μm in the simulation. Figure 9 illustrates the BL and MFA of the three-cladding fiber when *d_1_* is 16.5 μm, 17 μm, 17.5 μm, and 18 μm, respectively.

As shown in Figure 9a, the BL goes down gradually with the *t*_1_ and *d*_1_, and about half of the BL values are greater than 1 × 10^−1^ dB/m, so the BL has been raised by two orders of magnitude compared with that in Figure 3a. From Figure 9b, it can be learned that the MFA remains unchanged with *d*_1_, and it is also not effectively affected with *t*_1_. Compared to the result in Figure 3b, the MFA in Figure 9b can be considered the same as it. When the number of claddings decreases, the BL will be increased. The reason is that the introduction of multiple refractive index claddings can improve the refractive index difference between the core and cladding, which contributes to the decrease in BL. Due to the long distance between the multi-cladding and the fiber core, the change in multi-cladding has almost no effect on the MFA. As a result, the multi-cladding can reduce the BL effectively, but has no effect on enlarging the MFA.

### 4.3. Importance of GRIR

**Figure 10:**
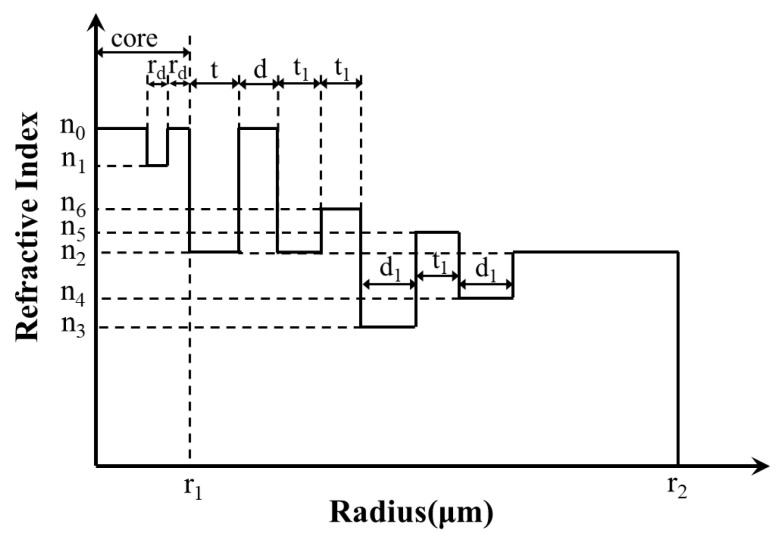
The RIP of the multi-cladding fiber with CIC and a step-refractive index ring.

**Figure 11:**
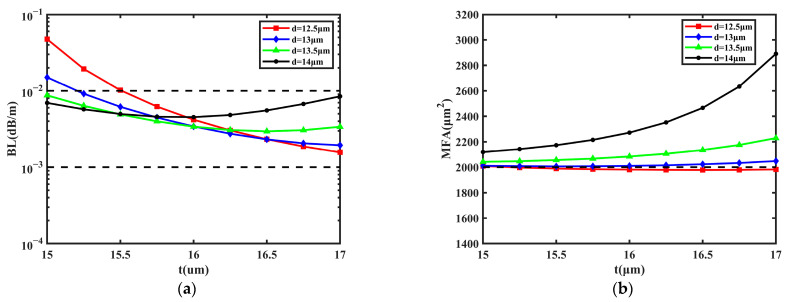
The influence on BL and MFA of the step-refractive index ring fiber with the change in *t* and *d*. (**a**) BL of FM; (**b**) MFA of FM.

Figure 10 shows the RIP of the CIC fiber with multi-cladding and a step-refractive index ring. Compared with the structure in Figure 1, the only difference is that the ring changes from a gradient-refractive index to a step-refractive index. The range of t is 15~17 μm and that of *d* is 12.5~14 μm in the simulation. In Figure 11, the change in BL and MFA are shown when *d* is 12.5 μm, 13 μm, 13.5 μm, and 14 μm, respectively. Because the fiber with a step-refractive index ring cannot support the transmission of the FM when *d* is 14.5 μm, the value 12.5 μm of *d* was added to the simulation of this part.

**Figure 12:**
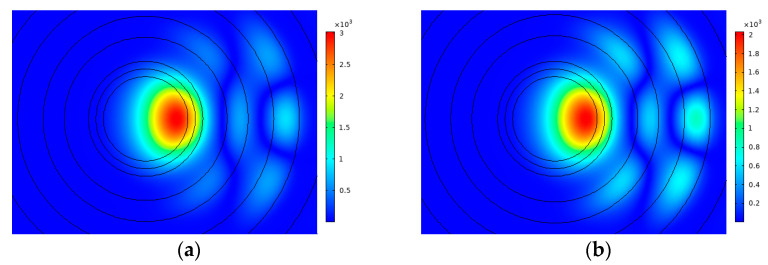
Mode field distribution of FM at (**a**) *t* = 16 and *d* = 14; (**b**) *t* = 17 and *d* = 14.

It can be learned from Figure 11a that the BL fluctuates irregularly, and most values are greater than 1 × 10^−3^ dB/m and less than 1 × 10^−2^ dB/m, so the result is close to that in Figure 4a. In Figure 11b, the MFA increases more and more quickly, because a larger and larger area of the FM leaks to the cladding instead of transmitting within the core with *t*. For example, under the condition that *d* is 14 μm, the mode field distribution of the FM is shown in Figure 12 when *t* is 16 μm and 17 μm. The phenomenon is not expected in the transmission of fiber. Additionally, most values of MFA in Figure 11b are close to 2000 μm^2^, except when *d* is 14 μm, and the largest MFA is 2891 μm^2^ when *d* is 14 μm and *t* is 17 μm. Compared with this, the MFA in Figure 4b can achieve 3232 μm^2^ at most, and the fiber can constrain FM transmission within the core well. As a conclusion, the GRIR has a significant effect on the MFA and supports the transmission of the FM better.

## 5. Bending Performance Research

The third and fourth sections describe the effect of structure on the BL and MFA. Section 5 introduces the bending performance of the proposed fiber, including the effect on bending radius and wavelength.

### 5.1. Bending Radius

**Figure 13:**
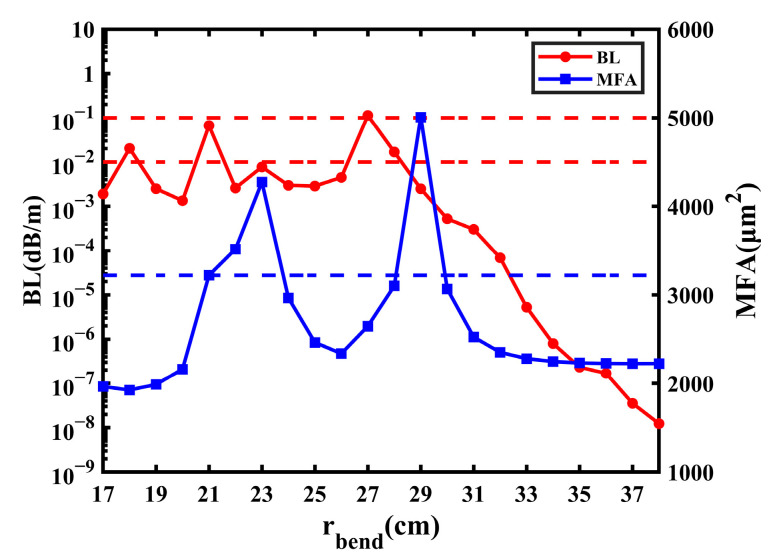
The influence on BL and MFA with the change in bending radius.

The simulation in the passage was carried out under the condition of a bending radius of 20 cm. In this part, the impact of other values of bending radius on the fiber performance is explored. Because the performance of fiber goes to steady when *r_bend_* is greater than 30 cm and the fiber cannot transmit the FM when *r_bend_* is less than 17 cm, the change in the BL and MF with *r_bend_* from 17 cm to 38 cm is studied in Figure 13. Due to the complexity of the proposed fiber, the modes are prone to change suddenly when the bending radius is small, which leads to the BL and MFA fluctuating irregularly. In Figure 13, it can be learned that the BL fluctuates with *r_bend_* from 17 cm to 27 cm, and then still decreases when *r_bend_* is greater than 27 cm; the lowest BL is 1.231 × 10^−8^ dB/m when *r_bend_* is 38 cm. Most values of BL are less than 1 × 10^−2^ dB/m, and there are only four values of it greater than 1 × 10^−2^ dB/m. The BL has approximately three peaks at an *r_bend_* of 18 cm, 21 cm, and 27 cm; the highest BL is 1.131 × 10^−1^ dB/m when *r_bend_* is 27 cm. When the BL is less than 1 × 10^−1^ dB/m, it is considered accepted. Therefore, when *r_bend_* is less than 30 cm, it can be considered that only when *r_bend_* is 27 cm can the BL be unacceptable.

**Figure 14:**
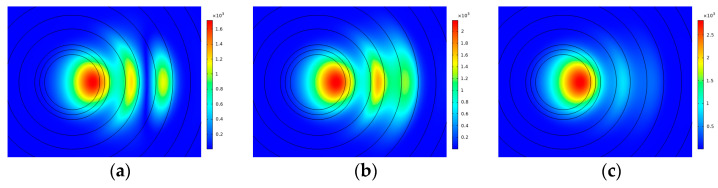
Mode field distribution of FM at (**a**) *r_bend_* = 23 cm; (**b**) *r_bend_* = 29 cm; (**c**) *r_bend_* = 31 cm.

Owing to the leaking of the FM into the cladding, some values of the MFA are abnormally large. The leakage of the FM is most severe at an *r_bend_* of 23 cm and 29 cm, the MFA achieves 4274 μm^2^ and 5007 μm^2^, respectively, and the mode field distribution of them is shown in Figure 14a,b. Additionally, the lowest MFA is 1925 μm^2^ when *r_bend_* is 18 cm. When *r_bend_* is greater than 30 cm, the performance of the proposed fiber goes steady, and the MFA finally remains about 2220 μm^2^ because the FM does not leak into the cladding but transmits within the core in general, which is shown in Figure 14c. When the MFA does not exceed 1000 μm^2^ of the steady state, the leakage is considered accepted. From Figure 13, when *r_bend_* is less than 30 cm, there are two ranges of MFA that are less than 3220 μm^2^; one is 17 cm to 21 cm, and the other is 24 cm to 28 cm.

In conclusion, the performance of the fiber is unsteady when *r_bend_* is smaller than 30 cm, but it can remain steady when the radius is greater than or equal to 30 cm. When *r_bend_* is less than 30 cm, there are two variations with low BL and leakage; one is an *r_bend_* of 17 cm to 21 cm, and the other is 24 cm to 28 cm (except for 27 cm).

### 5.2. Wavelength

**Figure 15:**
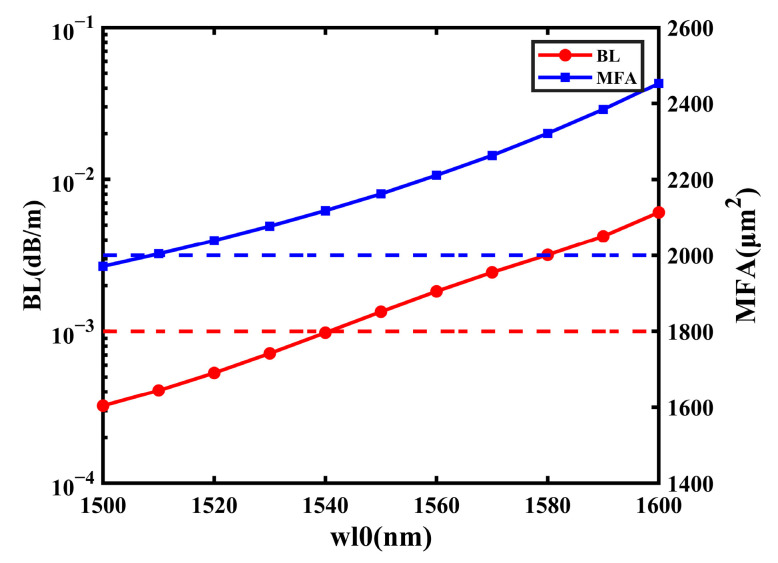
The influence on BL and MFA with the change in *wl0*.

The simulation analysis in the passage was carried out under the condition of an operating wavelength of 1550 nm. With the lowest transmitting loss, 1550 nm is the third window of optical communication and it is used most widely. However, the wavelength transmitted in practice is not fixed; the wavelength dependence is also an important factor to measure the optical fiber performance. As shown in Figure 15, both the BL and MFA increase steadily with wavelength. With the range of *wl0* from 1500 nm to 1600 nm, the highest BL is 6.072 × 10^−3^ dB/m, which is less than 1.000 × 10^−1^ dB/m. Thus, the change in wavelength has little effect on the BL of the proposed fiber. Additionally, only when *wl0* is 1500 nm can the MFA be less than 2000 μm^2^. Therefore, the analysis shows that the proposed fiber can maintain excellent performances of the BL and MFA when *wl0* changes from 1500 nm to 1600 nm.

## 6. Conclusions

In this paper, we propose an MClF with CIC and GRIR. The calculation and analysis of the relevant characteristics are under the condition that the bending radius is 20 cm and the wavelength is 1550 nm. In Section 3, the structural parameters are changed to explore the performance of the proposed fiber. It can be concluded that the MFA is always greater than 2000 μm^2^ and the BL is always less than 1.000 × 10^−2^ dB/m. The performance is best when *d* is 14.5 µm and *t* is 16.5 µm, corresponding to a BL of 8.452 × 10^−4^ dB/m and an MFA of 2010 µm^2^.

By the simulation, it can be concluded that the proposed fiber has outstanding performance with a low BL and large MFA. In Section 4, the importance of the three parts in the proposed fiber was explored separately. As a result, the CIC can reduce the BL and increase the MFA to some extent, and it can allow the smaller bending radius to transmit the FM. The multi-cladding can reduce the BL effectively, but does not affect enlarging the MFA. The GRIR has a more significant effect on the MFA than the BL, which can increase the MFA and support the transmission of the FM better. In Section 5, the bending performance research of the proposed fiber was discussed. When *r_bend_* is smaller than 30 cm, there are two variations with low BL and leakage; one is an *r_bend_* of 17 cm to 21 cm, and the other is 24 cm to 28 cm (except for 27 cm). Additionally, by analyzing the dependence on the wavelength of the incident light, it indicates that the fiber can maintain excellent performance when the wavelength changes by 100 nm. As a result, the proposed fiber can achieve a low BL and large MFA. The proposed fiber is significant for fiber in the home, high-power lasers, and it also can be used in optical communications.

## Figures and Tables

**Table 1:** Current status of similar research.

Type	BL (dB/m)	MFA (μm^2^)	Bending Radius (cm)
asymmetric PCF [27]	9.884 × 10^−5^	595.02	10
MCoF [28]	<1 × 10^−3^	>1400	50~60
SCF [14]	<1 × 10^−2^	914	20
CIF [25]	4 × 10^−2^	3110	17~20
helical cladding fiber [29]	1 × 10^−1^	2360	33
fiber with GRIR [26]	9.2 × 10^−2^	2622	20

## Data Availability

Not applicable.

## References

[1] Jain D., Jung Y., Nunez-Velazquez M., Sahu J.K. (2014). Extending single mode performance of all-solid large-mode-area single trench fiber. Opt. Express.

[2] Ning Y.Q., Chen Y.Y., Zhang J., Song Y., Lei Y.X., Qiu C., Liang L., Jia P., Qin L., Wang L.J. (2021). Brief Review of Development and Techniques for High Power Semiconductor Lasers. Act. Opt. Sin..

[3] Richardson D.J., Nilsson J., Clarkson W.A. (2010). High power fiber lasers: Current status and future perspectives. J. Opt. Soc. Am. B.

[4] Liu Y.H., Zhang F.F., Zhao N., Lin X.F., Liao L., Wang Y.B., Peng J.G., Li H.Q., Yang L.Y., Dai N.L. (2018). Single transverse mode laser in a center-sunken and cladding-trenched Yb-doped fiber. Opt. Express.

[5] Picozzi A., Millot G., Wabnitz S. (2015). Nonlinear optics: Nonlinear virtues of multimode fibre. Nat. Photonics.

[6] Beier F., Plotner M., Sattler B., Stutzki F., Walbaum T., Liem A., Haarlammert N., Schreiber T., Eberhardt R., Tunnermann A. (2017). Measuring thermal load in fiber amplifiers in the presence of transversal mode instabilities. Opt. Lett..

[7] Mitra P.P., Stark J.B. (2001). Nonlinear limits to the information capacity of optical fibre communications. Nature.

[8] Fini J.M. (2007). Intuitive modeling of bend distortion in large-mode-area fibers. Opt. Lett..

[9] Gao F.Y., Xu X.B., Song N.F., Li W., Zhu Y.H., Liu J.Q., Liang T.T. (2022). Low-Loss Isolated Anti-Resonant Core Photonic Bandgap Fiber. Chin. J. Lasers.

[10] Han J.L., Liu E.X., Liu J.J. (2019). Circular gradient-diameter photonic crystal fiber with large mode area and low BL. J. Opt. Soc. Am. A.

[11] Kabir S., Razzak S. (2018). An enhanced effective mode area fluorine doped octagonal photonic crystal fiber with extremely low loss. Photonic Nanostruct..

[12] Guo Z.J., Pei L., Ning T.G., Zheng J.J., Li J., Wang J.S. (2021). Resonant-ring assisted large mode area segmented cladding fiber with high-index rings in core. Opt. Commun..

[13] Pournoury M., Kim D. (2022). Bend-resistant octo-wing silica segmented cladding fiber with high index rings. Results Phys..

[14] Wang G.L., Ning T.G., Pei L., Ma S.S., Zhang J.C., Zheng J.J., Li J., Wei H., Xie C.J. (2020). A bending-resistant large mode area pixelated trench assisted segmented cladding fiber. Optik.

[15] Saitoh S., Takenaga K., Aikawa K. Demonstration of a Rectangularly-Arranged Strongly-Coupled Multi-Core Fiber. Proceedings of the IEEE 2018 Optical Fiber Communications Conference and Exposition.

[16] Xie Y.H., Pei L., Sun J.B., Zheng J.J., Ning T.G., Li J. (2019). Optimal design of a bend-insensitive heterogeneous MCF with differential inner-cladding structure and identical cores. Opt. Fiber Technol..

[17] Zhang Y., Jiang W.F., Chen M.Y. (2022). Design of ring-core few-mode multi-core fiber with low crosstalk and low benidng loss. Act. Opt. Sin..

[18] Suzuki K., Kubota H., Kawanishi S., Tanaka M., Fujita M. (2001). Optical properties of a low-loss polarization-maintaining photonic crystal fiber. Opt. Express.

[19] Zhang Y.Q., Lian Y.D., Wang Y.H., Wang J.B., Yang M.X., Luan N.N. (2021). Design and analysis of trench-assisted large-mode-field-area multi-core fiber with air-hole. Appl. Phys. B.

[20] Zhang Y.Q., Lian Y.D., Wang Y.H., Wang J.B., Yang M.X., Luan N.N., Lu Z.W. (2021). Study on dual-mode large-mode-area multi-core fiber with air-hole. Opt. Fiber Technol..

[21] Zhang Y.Q., Lian Y.D., Wang Y.H., Yang M.X., Wang J.B., Luan N.N., Wang Y.L., Lu Z.W. (2021). Design and analysis of trench-assisted dual-mode multi-core fiber with large-mode-field-area. Appl. Optics.

[22] Yang M.X., Lian Y.D., Wang J.B., Zhang Y.Q. (2019). Dual-Mode Large-Mode-Area Multicore Fiber with Air-Hole Structure. IEEE Photonics J..

[23] Hayashi T., Taru T., Shimakawa O., Sasaki T., Sasaoka E. (2011). Design and fabrication of ultra-low crosstalk and low-loss multi-core fiber. Opt. Express.

[24] Wang X., Lou S.Q., Lu W.L., Sheng X.Z., Zhao T.T., Hua P. (2015). Bend Resistant Large Mode Area Fiber with Multi-Trench in the Core. IEEE J. Solid-St. Circ..

[25] Miao X.F., Wu P., Zhao B.Y. (2019). Optimum design for a novel large mode area fiber with triangle-platform-index core. Mod. Phys. Lett. B.

[26] Tong Y., Chen S., Tian H.P. (2018). A bend-resistant low bending loss and large mode area two-layer core single-mode fiber with GRIR and multi-trench. Opt. Fiber Technol..

[27] She Y.L., Zhang W.T., Tu S., Liang G.L. (2021). Large mode area single mode photonic crystal fiber with ultra-low bending loss. Optik.

[28] Jin W.X., Ren G.B., Jiang Y.C., Wu Y., Xu Y., Yang Y.G., Shen Y., Ren W.H., Jian S.S. (2017). Few-mode and large-mode-area fiber with circularly distributed cores. Opt. Commun..

[29] Shen X., Li Y.Y., Yang T., Zheng J.J., Zhang Z.X., Wei W. (2022). Mode Transmission Characteristics of Heterogeneous Helical Cladding Large Mode Area Fiber. Act. Opt. Sin..

[30] Li Q.G., Wu W.W., Sun K.Y. (2013). Discussion on the Process of MCVD Gradient Index Multimode Fiber Prefabrication Rod. China Inst. Commun..

[31] Jain D., Alam S., Jung Y., Barua P., Velazquez M.N., Sahu J.K. (2015). Highly efficient Yb-free Er-La-Al doped ultra-low NA large mode area single-trench fiber laser. Opt. Express.

[32] Courant R.L. (1943). Variational Methods for the Solution of Problems of Equilibrium and Vibration. B Amer. Math Soc..

[33] Feng N.N., Zhou G.R., Xu C., Huang W.P. (2002). Computation of Full-Vector Modes for Bending Waveguide Using Cylindrical PerfectlyMatched Layers. IEEE J. Light. Technol..

[34] Rogier H., Zutter D.D. (2002). Berenger and Leaky Modes in Optical Fibers Terminated with a Perfectly Matched Layer. IEEE J. Lightwave Technol..

[35] Wang J., Pei L., Wang J., Ruan Z., Li J. (2021). Design and analysis for large-mode-area photonic crystal fiber with negative-curvature air ring. Opt. Fiber Technol..

[36] Zheng X.J., Ren G.B., Huang L., Zheng H.L. (2016). Study on bending losses of few-mode optical fibers. Acta Phys. Sin-Ch. Ed..

[37] Lee H., Ma T.Y., Mizuno Y., Nakamura K. (2018). Bending-loss-independent operation of slope-Assisted Brillouin optical correlation-domain reflectometry. Sci. Rep.-UK.

[38] Jain D., Sahu J.K. (2016). Large Mode Area Single Trench Fiber for 2 μm Operation. IEEE J. Light. Technol..

